# Mouse vascularized adipose spheroids: an organotypic model for thermogenic adipocytes

**DOI:** 10.3389/fendo.2024.1396965

**Published:** 2024-06-25

**Authors:** Laura Ingeborg Davidsen, Carolina E. Hagberg, Victor Goitea, Stine Meinild Lundby, Steen Larsen, Morten Frendø Ebbesen, Natasha Stanic, Hande Topel, Jan-Wilhelm Kornfeld

**Affiliations:** ^1^ Functional Genomics and Metabolism Research Unit, Institute of Biochemistry and Molecular Biology, Faculty of Science, University of Southern Denmark, Odense, Denmark; ^2^ Division of Cardiovascular Medicine, Department of Medicine Solna and Center for Molecular Medicine, Karolinska Institutet, Stockholm, Sweden; ^3^ Novo Nordisk Foundation Center for Adipocyte Signaling (ADIPOSIGN), University of Southern Denmark, Odense, Denmark; ^4^ Xlab, Center for Healthy Aging, Department of Biomedical Sciences, Faculty of Health and Medical Sciences, University of Copenhagen, Copenhagen, Denmark; ^5^ Danish Molecular Biomedical Imaging Center (DaMBIC), Institute of Biochemistry and Molecular Biology, Faculty of Science, University of Southern Denmark, Odense, Denmark

**Keywords:** adipose tissue microenvironment, 3D spheroids, vascularization, metabolism, crosstalk, oxygraph, browning, thermogenesis

## Abstract

Adipose tissues, particularly beige and brown adipose tissue, play crucial roles in energy metabolism. Brown adipose tissues’ thermogenic capacity and the appearance of beige cells within white adipose tissue have spurred interest in their metabolic impact and therapeutic potential. Brown and beige fat cells, activated by environmental factors like cold exposure or by pharmacology, share metabolic mechanisms that drive non-shivering thermogenesis. Understanding these two cell types requires advanced, yet broadly applicable *in vitro* models that reflect the complex microenvironment and vasculature of adipose tissues. Here we present mouse vascularized adipose spheroids of the stromal vascular microenvironment from inguinal white adipose tissue, a tissue with ‘beiging’ capacity in mice and humans. We show that adding a scaffold improves vascular sprouting, enhances spheroid growth, and upregulates adipogenic markers, thus reflecting increased adipocyte maturity. Transcriptional profiling via RNA sequencing revealed distinct metabolic pathways upregulated in our vascularized adipose spheroids, with increased expression of genes involved in glucose metabolism, lipid metabolism, and thermogenesis. Functional assessment demonstrated increased oxygen consumption in vascularized adipose spheroids compared to classical 2D cultures, which was enhanced by β-adrenergic receptor stimulation correlating with elevated β-adrenergic receptor expression. Moreover, stimulation with the naturally occurring adipokine, FGF21, induced *Ucp1* mRNA expression in the vascularized adipose spheroids. In conclusion, vascularized inguinal white adipose tissue spheroids provide a physiologically relevant platform to study how the stromal vascular microenvironment shapes adipocyte responses and influence activated thermogenesis in beige adipocytes.

## 1 Introduction

Adipose tissue (AT) is amongst the most dynamic organs, constantly expanding or shrinking based on metabolic status. Excessive amounts of white AT (WAT) is associated with obesity-related morbidities, while brown AT (BAT) acts as a thermogenic organ that may counteract the obese phenotype by acting as ‘nutrient sink’ upon cold exposure or pharmacological activation (1). Since confirming the presence of active BAT in adult humans, interest has surged in understanding the metabolic impact and therapeutic potential of thermogenic processes in BAT (2–5). Further, the inducible presence of beige or brite (brown in white) adipocytes within WAT depots, especially in the inguinal WAT (iWAT) region of rodents, alongside classical brown adipocytes in dedicated BAT depots like the interscapular region in both rodents and infants, has increased this interest (6, 7).

In mammals, both brown and beige thermogenic fat cells regulate systemic energy homeostasis, sharing similar thermogenic, morphological and biochemical characteristics (7–9). The ‘browning’ of white fat is induced by cold exposure, exercise, and treatment with peroxisome proliferator-activated receptor (PPAR)-γ- or β3-adrenergic receptor agonists (10–12). These activators induce traditional non-shivering thermogenesis, primarily via sympathetic nervous system (SNS) signaling, and discharge of catecholamine at nerve termini which ultimately bind and activate β3-adrenergic receptors. This results in transcriptional activation of the uncoupling protein 1 (*Ucp1*) gene, and translocation of UCP1 proteins into the inner mitochondrial membrane causes dissipation of the mitochondrial proton gradient, ultimately converting the energy from NADH/FADH_2_ oxidation into heat instead of ATP (1, 13).

Mature, lipid-laden adipocytes can be difficult to study *in vitro* due to their buoyancy, which makes their attachment to 2D cell culture plates challenging (14). In addition, to effectively study beige and brown adipocyte biology and their clinical implications requires tissue-engineered *in vitro* models that recapitulate the *in vivo* microenvironment or the so-called ‘adipose tissue niche’ (14, 15).

Mature adipocytes reside within a stromal vascular (SV) microenvironment comprising adipose mesenchymal stem cells and progenitors, neuronal cells, fibroblasts, immune cells and endothelial cells (16, 17). Furthermore, the extracellular matrix (ECM) provides a structural scaffold to facilitate adipocyte differentiation and growth signaling (14). Adipogenesis and angiogenesis are closely intertwined processes in spatial and temporal terms (18–20). Proximity of adipocytes to vasculature and neurite projections influences adipocyte maturation (16). WAT, and particularly BAT, are highly innervated by the SNS, triggering lipolysis and thermogenesis through norepinephrine release (21). Angiogenesis plays a pivotal role in modulating AT mass and metabolism (22). Optimal thermogenic function and enhanced ‘browning’ rely on proangiogenic factors like vascular endothelial growth factor (VEGF)-A. For example, ectopically overexpressing VEGF-A enhances vascularization, induces ‘browning’ of WAT, and protects against diet-induced obesity and metabolic dysfunction (23). Similarly, in BAT, VEGF-A overexpression increases vascularization and thermogenesis during chronic cold exposure, and protects against metabolic dysfunction induced by high-fat diet (24). On the other hand, macrophages are part of the hematopoietic niche of the SV fraction (SVF) in the AT (16). Initially, it was postulated that alternatively activated AT macrophages constitute a source of catecholamines, thereby directly activating beige thermogenesis (25). However, contradictory reports indicated that AT macrophages play a role in the bioavailability rather than representing a biologically meaningful source of norepinephrine (26–28). Further, thermogenically activated BAT secretes C-X-C motif chemokine ligand-14 (CXCL14), a newly reported regulatory chemokine that recruits alternative activated AT macrophages, promoting the browning of WAT (29). Overall, the microenvironment significantly influences the maturation of brown and beige adipocytes. Intricate crosstalk among cells in the adipose niche is crucial for regulating thermogenic fat activation. The heterogeneity of the SVF allows for the possibility of incorporating different cell types in an adipose model thus more closely reflecting the SV microenvironment of the tissue *in vivo* (30).

Unlocking the therapeutic potential of brown and beige AT requires technological advancements that account for the great variety of hormonal and cellular factors influencing the cellular composition and vascularization of the AT niche (31). The vasculature of BAT is pivotal in non-shivering thermogenesis, making BAT one of the most highly vascularized tissues, with an extensive network of microvessels (19, 32). Recent developments in bioengineered *in vitro* models of AT facilitate important cell-cell and cell-ECM interactions, making them superior at recapitulating the native *in vivo* milieu compared to standard 2D cultures of SVF-derived *in vitro* differentiated adipocytes (30). 3D engineered *in vitro* models of functional beige AT can be used to study metabolic conditions, identify therapeutic targets, and evaluate treatment strategies (33). Although these models are in early stages of development, current models fail to recreate the structural and functional complexity of beige AT, including mitochondrial biogenesis, a vascular network and, importantly, responsiveness to thermogenic hormones such as fibroblast growth factor (FGF) 21 (33). 3D spheroids of human WAT derived from the subcutaneous SVF have shown that endothelial cells are able to self-assemble into highly organized vascular networks (34, 35). These models however have not been used to study beige and brown adipocytes yet.

As an alternative to 2D cultures, 3D cultures represent the cellular crosstalk within a complex metabolic organ, such as the AT, in a more physiological fashion, are less invasive compared to preclinical animal models and excel by high reproducibility. Moreover, studying the microenvironment in 3D facilitates release of specific soluble factors and modulation of cell-matrix and cell-cell interactions (15). We, here, present vascularized adipose spheroids of mouse iWAT as an easy-to-follow, universal platform for studying the AT SV microenvironment. The appearance of adipocyte capillary structures and vessel formation not only mimics the cellular heterogeneity within the adipose tissue in a scalable 3D setup but also enhances expression of genes associated with adipogenesis, oxidative phosphorylation, beta-adrenergic thermogenesis, and thereby more faithfully reflect the gene-regulatory and metabolic properties of *in vivo* adipocytes compared to routinely used 2D systems.

## 2 Materials and methods

### 2.1 Mice

Six to eight weeks old C57/BL6 male mice were purchased from Taconic (Denmark). All mice were housed in a temperature-controlled environment with a 12-hour light-dark cycle and had ad libitum access to food and water. Mouse experiments were conducted with permission from the Danish Animal Experiments Inspectorate, reference number: 2023–15-0201–01544.

### 2.2 Stromal vascular fraction isolation

Inguinal white adipose tissue was dissected, minced, and digested in Ham’s F12 medium (Merck, F4815) supplemented with collagenase II (Worthington) and DNAse (Sigma, 10104159001). It was then incubated in a 37°C shaker for 30–40 min and afterwards the digested tissue was filtered through a 300 μm pluristrainer (pluriSelect Life Science, 43–50300). To the filtered tissue, 20 mL of Ham’s F12 medium supplemented with 0.1% Biotin/D-Pantothenate (33mM/17mM) (Sigma, B-4639 and P5155), 1% penicillin-streptomycin (Lonza, DE17/602E) and 10% FBS (Sigma, F7524) was added, in the following referred to as complete medium. After two rounds of centrifugation at 300 rcf for 5 min each, the pellet was resuspended in 10 mL complete medium and centrifuged for another 5 min at 500 rcf. The supernatant was discarded, and the pellet was resuspended in complete medium, followed by centrifugation for 5 min at 500 rcf. To remove erythrocytes, the pellet was resuspended in 1 mL of erythrocyte lysis buffer (Qiagen) for 5 min followed by additional 9 mL of complete medium and centrifuged for 5 min at 500 rcf. Finally, the pellet was resuspended in complete medium with the addition of 20% FBS and filtered through a 70 μM pluristrainer (pluriSelect Life Science, 43–50070). Cells were immediately used for respective experiments after counting and were cultured at 37°C with 5% CO_2_, unless otherwise stated.

### 2.3 2D monolayer culture of the inguinal stromal vascular fraction

The isolated inguinal white adipose tissue stromal vascular fraction was resuspended in Preadipocyte Basal Medium (BulletKit, Lonza, PT-8002) with the addition of L-glutamine, GA-1000 and FBS (SingleQuots, part of BulletKit, Lonza, PT-8002) and seeded in 12–24-wells (Thermo Scientific, 150628 and 142475) depending on the experiment. Upon reaching confluency, adipogenesis was induced using Preadipocyte Growth Medium (BulletKit, Lonza, PT-8002) with the addition of insulin, dexamethasone, IBMX and indomethacin (SingleQuots, part of BulletKit, Lonza, PT-8002). The medium was exchanged every other day with diluted Preadipocyte Growth Medium and full differentiation was achieved seven days after induction of adipogenesis.

### 2.4 Vascularized adipose spheroids of the inguinal stromal vascular fraction

This protocol builds on previous work published by (35) for human subcutaneous white adipose tissue. The isolated inguinal white adipose tissue stromal vascular fraction was resuspended in Endothelial Growth Medium (BulletKit, Lonza, CC-3162) with the addition of FBS, hydrocortisone, FGF, VEGF, IGF, ascorbic acid, EGF, GA-1000 and heparin (SingleQuots, part of BulletKit, Lonza, CC-3162), and seeded at approximately 10.000 cells per 200 μL in each well of a 96-well ultra-low attachment plate with round bottom (Thermo Scientific, 15227905). To maintain high humidity, 200 μL PBS was added to the outer wells of the plate. If a single large spheroid did not form in each well by day three, the plate was centrifuged at 500–600 rcf for 5 min to enhance cell aggregation. On day six, 150 μL of the Endothelial Growth Medium was discarded from each well, and the spheroids were embedded in 4 mg/mL growth factor reduced Matrigel (Corning, 356231). Matrigel polymerization took place for 30–60 min at 37°C, followed by the addition of 100 μL Endothelial Growth Medium to each well. Adipogenesis was induced on day 10 by replacing 100 μL of the Endothelial Growth Medium with an equal amount of Preadipocyte Growth Medium (BulletKit, Lonza, PT-8002) with the addition of L-glutamine, GA-1000, FBS, insulin, dexamethasone, IBMX and indomethacin (SingleQuots, part of BulletKit, Lonza, PT-8002). At day 15, 50 μL diluted Preadipocyte Growth Medium was added to each well, and on day 20, 150 μL of the Preadipocyte Growth Medium was exchanged with new diluted Preadipocyte Growth Medium. Unless otherwise specified, all spheroids were harvested on day 21.

### 2.5 β-adrenergic stimulation of inguinal adipocytes

To induce thermogenesis, 2D cultures and vascularized adipose spheroids were either stimulated with the pan β-adrenergic agonist Isoproterenol (Sigma, I5627) for 6 hours (final conc. 1 μM) or the browning inducer FGF21 (Recombinant Human FGF21, PeproTech, 100–42) for 48 hours (final conc. 50 mM).

### 2.6 Depolymerization and harvesting of the spheroids

For both 2D cultures and vascularized adipose spheroids the medium was discarded. Cells cultured in 2D were allowed to detach on a plate shaker in 500 μL Trizol. For spheroids, cell recovery solution (Corning, 354253) was added to depolymerize the Matrigel at 4°C for 30–60 min. For immunofluorescence analysis, the depolymerized Matrigel was discarded, and PBS was added until further processing. For RNA isolation, 5–10 spheroids were pooled in FastPrep24 tubes. The FastPrep24-tubes were then centrifuged at 4°C for 5 min at 1300 rpm, and the supernatant was discarded. Before homogenization using FastPrep-24 5G (MP Biomedicals), 500 μL Trizol was added to the tubes. All samples were stored at -80°C except for those processed for immunofluorescence staining.

### 2.7 Immunofluorescence staining

The isolated spheroids were fixed in 4% PFA at room temperature (RT) for 30 min, followed by three 5-min washes with PBS. Next, the fixed spheroids were permeabilized and blocked to inhibit non-specific binding using a solution of 10% saponin (Sigma, S7900–25G) and 5% BSA (Sigma, 126579) in PBS for 1 hour at RT. After two 5-min washes with PBS, the spheroids were stained overnight at RT with primary CD31 antibody (BD Biosciences, 550274) to visualize endothelial cells, Bodipy (final conc. 1.5 μM, kindly provided by DaMBIC) for lipids and Hoechst (final conc. 3mg/L, Invitrogen, H3570) for nuclei in 1% BSA and 0.1% Tween in PBS. The following day, the spheroids underwent three 5-min washes with PBS and were incubated for 2 hours at RT with Alexa Fluor 647 Goat Anti-Mouse secondary antibody (Invitrogen, A21247), diluted in 1% BSA and 0.1% Tween in PBS. After three 5-min washes with PBS, the stained spheroids were prepared for mounting. Mounting was achieved using double-sided nano gel tape with pre-laser cut wells on SuperFrost-Plus slides (VWR, 631–0108). Each well accommodated one spheroid, mounted with a solution of 90% glycerol (Sigma-Aldrich, G5516) and Propyl Gallate (Sigma, P3130) in 1xPBS and finished with a cover glass (VWR, 631–1572) placed on top.

### 2.8 Bioimaging, microscopy and image acquisition

The morphology of the vascularized adipose spheroids, including vascular sprouting, was examined using a Nikon Eclipse Ts2 Inverted light microscope. Measurements of the total spheroid surface area was conducted on light microscopy images using Fiji ImageJ software (v2.14.0). The immunofluorescently stained 3D spheroids were examined via confocal microscopy using a Nikon A1R on a Ti-2 microscope body. The following laser wavelengths were used: 405 nm (Hoechst), 488 nm (Bodipy) and 640 nm (CD31). Light settings, laser intensity and detector gain were adjusted according to each spheroid condition. Post-acquisition analysis of the confocal microscopy images was carried out using Fiji ImageJ software.

### 2.9 RNA purification

Total RNA extraction was performed using the chloroform extraction method. Briefly, 100 μL chloroform was added to the samples, the tubes were vortexed, and then centrifuged at 4°C for 10 min at 10.000 rcf. The upper aqueous phase was carefully transferred to new tubes, and 300 μL of ethanol 96% was added. After having been vortexed and spun down, the suspension was loaded into EconoSpin columns (Epoch Life Science, 1920). The columns were centrifuged at 13.000 rcf for 30 sec followed by three washes with 500 μL RPE buffer, each time centrifuged at 13.000 rcf for 30 sec. Finally, the columns were allowed to spin dry at 13.000 rcf for 2 min before transferring them to new tubes. Elution of RNA was performed using DEPC H_2_O via centrifugation at 16.000 rcf for 1 min. RNA concentration was immediately quantified afterwards using the LVis microplate for RNA on the ClarioStar (BMG Labtech) microplate reader and MARS data analysis software.

### 2.10 Reverse transcription and qPCR

RNA was reverse transcribed into cDNA using the High-Capacity cDNA Reverse Transcription Kit (Applied Biosystems, 4368814) following the manufacturer’s instructions. The cDNA was amplified in a Bio-Rad CFX Opus 384 (Bio-Rad Laboratories, Inc.) qPCR machine in 384 well format using 4 μL diluted cDNA, 0.5 μL mix of gene-specific forward and reverse primers (5 μM each), and 4.5 μL FastStart Essential cDNA Green Master (Roche). The temperature protocol included 45 cycles of 15 sec at 95°C, 45 sec at 60°C and 45 sec at 72°C. All primer-sample combinations were run in duplicates, and Cq values were calculated as the second derivative maximum. Genes of interest were normalized against a housekeeper gene using the delta Cq method. Primer sequences can be found in **Supplementary Table 1**.

### 2.11 High-resolution RNA sequencing, library construction and processing

The quality of the RNA was assessed via Fragment Analyzer (Advanced Analytical Technologies, Inc.) using the Standard Sensitivity RNA Analysis kit (DNF-489) and following the manufacturer’s protocol. RNA sequencing was carried out on the Illumina NovaSeq 6000 platform, as per the manufacturer’s instructions (NEBNext Ultra II RNA Library Prep Kit for Illumina, NEB #E7775), using 200 ng of total RNA for cDNA library preparation.

The quality control pipeline for raw sequencing reads encompassed a comprehensive suite of tools and methodologies. Initially, an assessment of read quality was conducted using FastQC (v0.11.9), FastQ_Screen (v0.15.1) and AdapterRemoval (v2.3.2). Subsequently, raw sequencing reads were aligned to the mouse genome (mm39) employing STAR (v2.7.9a). Various mapping quality control steps were undertaken, including the generation of statistics, index statistics, and flag statistics using Samtools (Samtools v1.15.1). Additional quality metrics were obtained through CollectAlignmentSummaryMetrics, QualityScoreDistribution,MeanQualityByCycle, CollectInsertSizeMetrics, CollectRnaSeqMetrics, using Picard (v2.25.5) and by and bamqc using Qualimap (v2.2.1). Library complexity was evaluated using Preseq (v3.2.0) and PBC bottlenecking metrics which were computed using Bedtools (v2.30.0). Duplicates were identified but retained in the dataset using Picard MarkDuplicates. Filtering steps were applied to exclude reads with low mapping quality (MAPQ<30), reads/mates that were unmapped, reads failing platform and those not classified as primary alignments (samtools view -q 30 -F 780). For additional RNA-seq quality control, tools such as qualimap rnaseq (v2.2.1), rnaseqc (RNA-SeQC v2.4.2), and various metrics from RSeQC v4.0.0 (read_duplication, read_GC, inner_distance, junction_annotation, junction_saturation, bam_stat, infer_experiment, read_distribution, geneBody_coverage, and tin) were employed. To summarize the diverse quality metrics, an HTML report was generated using MultiQC (v.1.14). The quantification of read pairs in exonic regions was performed using featureCounts (Subread v2.0.3) with GENCODE vM33 GTF reference annotation.

Data analysis was conducted in R (v4.3.2), utilizing DESeq2 (v1.42) for normalization of raw count reads through the median of ratios method. Low count genes were filtered, retaining those with a total count greater than or equal to 10. Differential gene expression analysis was performed using DESeq2. Only genes with a *p*-adjusted value < 0.05 and a fold change > 1.5 were considered as differentially expressed genes (DEGs). To visually represent the relationships between experimental conditions, Principal Component Analysis (PCA) was applied, while a volcano plot was generated to depict upregulated vs downregulated genes. Functional analysis included Overrepresentation Analysis (ORA) of DEGs and Gene Set Enrichment Analysis (GSEA) of expressed genes ranked by Log2FoldChange (3Dvs2D). The Kyoto Encyclopedia of Genes and Genomes (KEGG) database and Molecular Signatures Database (MSigDB) Hallmarks were employed for these analyses. DEGs resulting from multiple comparisons (3Dvs2D * with/without Isoproterenol) were subjected to hierarchical clustering by the Ward D2 method and aggregated into 6 kmeans clusters. The optimal number of clusters was determined by evaluating the results of the Elbow, Silhouette, and Gap statistic methods. Additionally, the NbClust function from the NbClust package (v3.0.1) was utilized, applying 30 indices to suggest the best number of clusters. A heatmap representing the 6 clusters was generated using pheatmap (v1.0.12). GSEA functional analysis was then applied to these clusters as previously described, providing deeper insights into the functional patterns within the identified gene clusters.

### 2.12 High-resolution respirometry via OROBOROS technology

The isolated inguinal white adipose tissue stromal vascular fraction was counted when seeding the cells in either 12-well (2D) or 96-well ULA plate (vascularized adipose spheroids). Oxygen consumption was measured using a high-resolution oxygraph (Oroboros Oxygraph-2K, Austria). Oxygen concentration and rate of oxygen consumption were continuously recorded using DAT LAB software 6. Mature adipocytes cultured in 2D were trypsinized (Sigma, T4174), centrifuged at 500 rcf for 5 min, and the cell pellet resuspended in 2 mL Preadipocyte Basal Medium (BulletKit, Lonza, PT-8002, without supplementation of SingleQuots: FBS, GA-1000 and L-glutamine). Similarly, mature 3D spheroids were harvested and pooled in 2 mL Preadipocyte Basal Medium (BulletKit, Lonza, PT-8002, without supplementation of SingleQuots: FBS, GA-1000 and L-glutamine). The 2 mL cell suspension from each preparation was added to the oxygen electrode chambers, where it was magnetically stirred and kept at 37°C. Any air bubbles were removed before the chambers were closed. The cell suspensions were incubated in the oxygen electrode chambers until a stable basal respiratory rate was reached. After this, stimulations were successfully performed using a Hamilton syringe with PBS or Isoproterenol (final conc. 1 μM), oligomycin (final conc. 2.5 μM), FCCP (final conc. 0.5 μM) or Antimycin A (final conc. 2.5 μM). The oxygen consumption rates were expressed in pmol O_2_/(sec x mL) or represented as percentage of basal oxygen consumption rates.

### 2.13 Statistical analysis

Normalized gene counts and qPCR data were analyzed using unpaired, non-parametric *t* test followed by Mann-Whitney via GraphPad Prism software (v10.1.0) A *P* value of ≤ 0.05 was considered significant.

## 3 Results

### 3.1 Inguinal stromal vasculature-guided assembly into vascularized adipose spheroids

**Figure 1:**
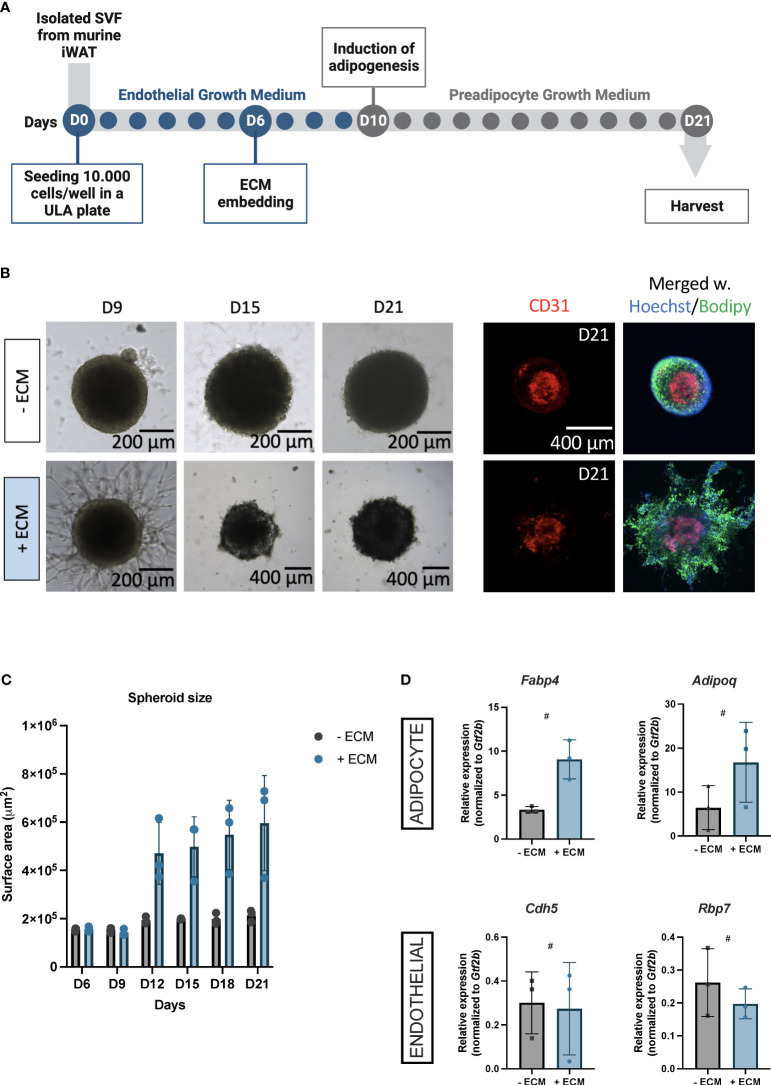
**(A)** Schematic workflow for constructing vascularized adipose spheroids from the inguinal white adipose tissue (iWAT) in mice. Isolated stromal vascular fraction (SVF) is seeded in an ultra-low attachment (ULA) plate in endothelial growth medium (blue dots). The spheroids are embedded in extracellular matrix (ECM) at day (D)6, adipogenesis is induced at D10 by exchanging the medium to preadipocyte growth medium (grey dots) and the spheroids are ready to use at day 21. **(B)** Light microscopy images for morphological assessment of spheroids embedded +/- ECM from D9, 15 and 21. Immunofluorescence staining for the endothelial marker CD31 (red) in spheroids +/- ECM at D21, which have been merged with counterstained nuclei (blue) using Hoechst, and Bodipy to identify lipids (green). Scalebars 200 and 400 μM. **(C)** Quantification of spheroid surface area at the indicated days. Each dot represents one spheroid, n = 3 per time point, per condition. **(D)** Relative mRNA expression of adipocyte markers, *Fabp4* and *Adipoq*, and endothelium markers, *Cdh5* and *Rbp7* in spheroids – ECM (grey) and + ECM (blue). n = 3 per condition. The mRNA expressions were normalized to *Gtf2b* expression and presented as means ± standard deviation (SD). Statistics were calculated using unpaired, non-parametric *t*-test followed by Mann-Whitney, # indicates *p* = 0.1.

We aimed to establish universal and versatile vascularized adipose spheroids assembling the iWAT SVF from mice. We hypothesized that embedding the spheroids in an ECM scaffold would enhance the vascularization based on published reports (35).The workflow of our model (**Figure 1A**) is based on isolated SVF from iWAT seeded in Endothelial Growth Medium (EGM) in an ultra-low attachment plate. The cells formed aggregates and were embedded in ECM (Matrigel) at day (D) six. At D10, half of the EGM was replaced with Preadipocyte Growth Medium (PGM), fostering adipogenesis for 11 days before harvesting the spheroids on D21.

Initially, we explored whether Matrigel was essential for the assembly of spheroids into an organotypic vascularized adipose niche as adipogenesis and angiogenesis are interconnected processes (17, 19, 36, 37) and both adipocytes and adipose-derived endothelial cells synthesize and secrete ECM components themselves (38). Morphologically, vascular sprouting appeared three days after Matrigel-embedding, unlike the spheroids without Matrigel, showing no sprouting (**Figure 1B**, D9). Fluorescent staining for the endothelial cell marker cluster of differentiation (CD) 31 indicated CD31^+^ endothelial cells in the spheroid centers both with and without Matrigel (**Figure 1B**, D21), suggesting that endothelial cells within spheroids without Matrigel were likely quiescent as these did not induce vascular sprouting. Likewise, Bodipy-labeled lipid droplets were detectable in both spheroids (**Figure 1B**, D21), reflecting that the adipocytes were mature.

Interestingly, we observed an overall increase in spheroid growth when embedded in Matrigel (**Figure 1B**, D15 and D21), which was confirmed by surface area measurements (**Figure 1C**), emphasizing the cellular expansion of multi/mono-locular adipocytes beyond what is seen in 2D setups. Additionally, Matrigel-embedded spheroids showed increased expression of adipocyte maturity genes (i.e. *Fabp4* (Fatty acid binding protein 4) and *Adipoq* (Adiponectin)) (**Figure 1D**), which is consistent with results from human subcutaneous white adipose vascularized spheroids (35). Endothelial mRNA expression of *Cdh5* (Cadherin 5) and *Rbp7* (Retinol binding protein 7) showed endothelial mRNA expression in both types of spheroids (**Figure 1D**), consistent with our immunofluorescence staining.

Thus, we have here established a mouse vascularized adipose spheroid model based on SVF from thermogenic iWAT. Our findings demonstrate that ECM-embedding supports outgrowth of vascular structures and, on the other hand, allow for adipogenic differentiation.

### 3.2 The inguinal niche reveals enhanced induction of metabolic genes in spheroids

**Figure 2:**
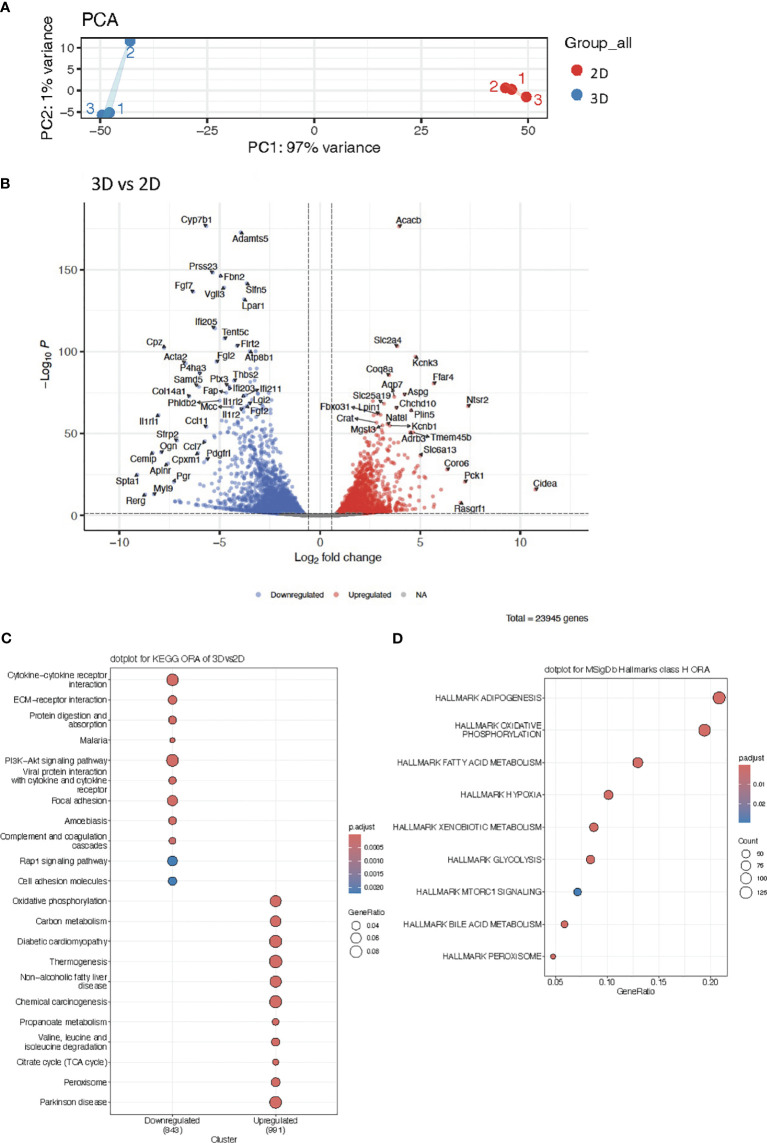
**(A)** Principal Component Analysis (PCA) plot of RNA sequencing data from 2D cultures and vascularized adipose spheroids (3D). **(B)** Volcano plot of differentially expressed genes (DEG) in 3D vs 2D cultures. **(C)** KEGG Overrepresentation Analysis (ORA) dotplot of enriched pathways in upregulated and downregulated genes of 3D vs 2D cultures. **(D)** MSigDb ORA dotplot of enriched pathways in upregulated genes of 3D spheroids. n = 3. Only genes with a *p*-adjusted value of < 0.05 and a fold change > 1.5 were considered as DEG.

We next asked whether vascularized adipose spheroids were better at reflecting the inguinal cellular composition and metabolic processes leading to a more adipogenic microenvironment and thus we performed RNA sequencing to compare transcriptional profiles between the vascularized adipose spheroids and conventional *in vitro* differentiated 2D cultures (**Figure 2**).

We conducted Principal Component Analysis (PCA) to visualize the complexity of our data and allow group-level comparison. The PCA delineated a clear separation between the two culturing methods (2D, 3D), with PC1 explaining 97% of the variance (**Figure 2A**), highlighting the strong differences between the 2D cultures and vascularized adipose spheroids. Subsequently, differential expressed gene (DEG) analysis revealed 23.945 genes regulated between conditions (**Figure 2B**). Within the vascularized adipose spheroids, upregulated genes were predominantly connected to metabolic pathways, such as *Slc2a4* (Solute carrier family 2 member 4, also known as Glucose transporter 4) essential for insulin-stimulated glucose uptake, and *Pck1* (Phosphoenolpyruvate carboxykinase 1), pivotal in gluconeogenesis and lipid metabolism (39–41). Additional genes like *Plin5* (Perilipin 5), facilitate triglyceride storage and suppress insulin resistance (42), and *Crat* (Carnitine acetyltransferase) transports fatty acids for β-oxidation across the mitochondrial membranes (43), showed elevated expression, emphasizing that the vascularized adipose spheroids had a higher induction of metabolic genes. Intriguingly, upregulation of the β-adrenergic receptor (*Adrb*)*3* in the vascularized adipose spheroids, suggested that the spheroids may serve as a more reliable and catecholamine-responsive system for studying thermogenesis, despite the absence of SNS innervation. Conversely, downregulated genes in the vascularized adipose spheroids compared to 2D were linked to ECM formation and cell signaling such as *Fbn2* (Fibrillin 2)*, Thbs2* (Thrombospondin 2), *Col14a1* (Collagen type XIV alpha 1 chain), and immune responses such as *Ifi205* (Interferon activated gene 205*)* and *Ccl11* (CC motif chemokine ligand 11), potentially reflecting induction of endogenous ECM production in 2D.

Next, we applied Kyoto Encyclopedia of Genes and Genomes Over-Representation Analysis (KEGG ORA) to identify which specific biological pathways were associated with DEGs and to reveal whether certain biological pathways were more affected by the experimental conditions than would be expected by random chance. KEGG ORA unveiled enriched pathways in the upregulated genes, encompassing oxidative phosphorylation, carbon metabolism, thermogenesis, and the citric acid cycle (TCA) cycle (**Figure 2C**). Conversely, downregulated genes were associated with cytokine-cytokine receptor interaction, ECM-receptor interaction, PI3K-Akt (phosphoinositide-3-kinase–protein kinase B) signaling, and focal adhesion (**Figure 2C**), again highlighting an increased expression of metabolic genes in the vascularized adipose spheroids compared to 2D.

Further investigation into underlying biological pathways utilizing Molecular Signatures Database (MSigDb) Hallmarks ORA, a bioinformatic tool (44) to assess whether curated gene sets of biological states or processes (Hallmark) from MSigDb were significantly over-represented in our DEGs, affirmed that upregulated genes predominantly cluster in categories like adipogenesis, oxidative phosphorylation, fatty acid metabolism and glycolysis (**Figure 2D**).

### 3.3 Comparative transcriptional profiling of vascularized adipose spheroids and 2D cultures

**Figure 3:**
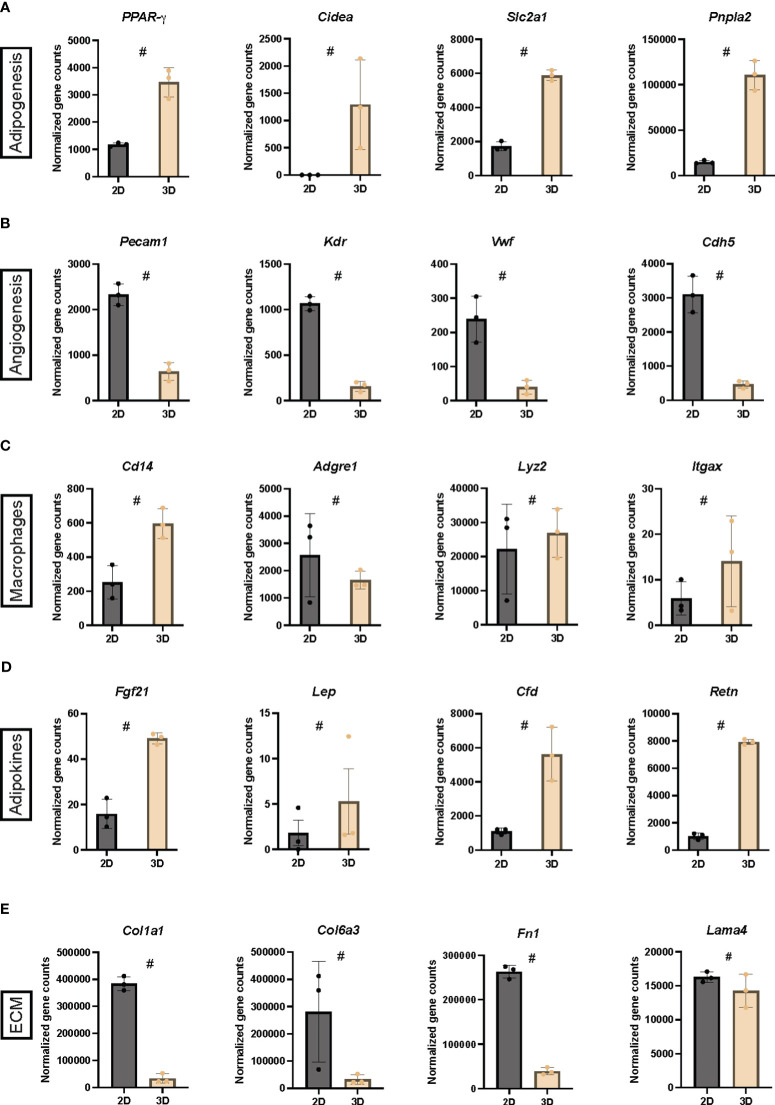
**(A-E)** Normalized gene counts derived from the RNA sequencing belonging to five cellular and functional categories, adipogenesis, angiogenesis, macrophages, adipokines and extracellular matrix (ECM) in 2D cultures and vascularized adipose spheroids (3D). n = 3. Data is presented as means ± standard deviation (SD), except for *Lep*, which is presented as means ± standard error of mean (SEM). Statistics were calculated using unpaired, non-parametric *t*-test followed by Mann-Whitney, # indicates *p* = 0.1.

Intrigued by the findings in **Figure 2**, we selected four genetic markers from the normalized gene counts representing five cellular and functional categories: adipogenesis, angiogenesis, macrophages, adipokines, and ECM (**Figure 3**). As expected, an increased expression of adipogenic markers was observed when comparing 2D cultures and vascularized adipose spheroids (**Figure 3A**). Notably, *Ppar-γ*, a key regulator of adipogenesis, *Atgl* (Adipose triglyceride lipase), a rate-limiting enzyme for release of FFAs during intracellular lipolysis, and *Slc2a1* (Solute carrier family 2 member 1, also known as Glucose transporter 1), ubiquitously expressed and vital for adipose glucose uptake (45) showed increased expression in the vascularized adipose spheroids compared to 2D cultures. Intriguingly, *Cidea* (Cell death inducing DFFA like effector A), a gene predominantly expressed in BAT and thought to regulate UCP1 activity (46), was upregulated in the vascularized adipose spheroids. Cidea is induced by PRDM16, and its expression is considered indicative of the thermogenic potential of adipocytes in relation to the regulation of energy expenditure (46, 47).

Surprisingly, a general increase in endothelial markers was observed in the 2D cultures compared to the vascularized adipose spheroids (**Figure 3B**). This was unexpected since, during the first 10 days of spheroid culture, EGM was added, while 2D cultures used only PGM. Despite this, *Pecam1* (Platelet endothelial cell adhesion molecule 1, also known as CD31) expression was markedly increased in the 2D cultures, however immunofluorescence staining revealed the presence of CD31^+^ endothelial cells in the vascularized adipose spheroids too (**Figure 1B**). Furthermore, the expression of macrophage markers showed no clear difference between the two culture methods (**Figure 3C**). *Itgax* (Integrin alpha X, also known as CD11c), upregulated in the vascularized adipose spheroids, and *Adgre1* (Adhesion G protein-coupled receptor E1, also known as Emr1 or F4/80), upregulated in the 2D culture, are expressed on mouse dendritic cells and a subpopulation of monocytes/macrophages. Both are associated with an M1-like macrophage phenotype during high fat diet-induced obesity (48, 49). Notably, *Lyz2* (Lysozyme 2), mainly expressed in macrophages and only modestly upregulated in the vascularized adipose spheroids, has been shown to be increased in iWAT mouse models of genetic and diet-induced obesity and is negatively correlated with various adipocyte-related genes (50). Another macrophage marker increased in the vascularized adipose spheroids was *Cd14*, expressed in both macrophages and myeloid cells.

Adipocytes express and secrete hormones also known as adipokines, which act locally (autocrine/paracrine) and in circulation (endocrine) (51). *Lep* (Leptin), *Cfd* (Adipsin), and *Retn* (Resistin), were upregulated in the vascularized adipose spheroids (**Figure 3D**). Additionally, *Fgf21* (Fibroblast growth factor 21), an adipokine primarily produced by the liver, but with an additional, cell-intrinsic role in browning of WAT was also upregulated in the spheroids (52).

Like the angiogenic markers shown in **Figure 3B**, ECM markers (**Figure 3E**) were upregulated exclusively in the 2D cultures compared to the vascularized adipose spheroids. During adipogenesis, the ECM evolves from a fibrillar to a laminar structure, and mature adipocytes spend substantial amounts of chemical energy on ECM maintenance, a process regulated by insulin, energy metabolism, and mechanical forces (53). Core proteins of the adipocyte ECM (53, 54), such as *Col1a1* (Collagen type I alpha 1 chain), *Col6a3* (Collagen type VI alpha 3 chain), *Fn1* (Fibronectin 1), and *Lama4* (Laminin subunit alpha 4), were all increased in the 2D cultures, highlighting the potential endogenous ECM production in the 2D cultures.

Thus, our results showed upregulation of endogenous ECM and endothelial genes in 2D cultures, whereas the vascularized adipose spheroids showed transcriptional upregulation of adipogenic and thermogenic genes, indicating that adipocytes within the spheroids were more mature.

### 3.4 β-AR activation of vascularized beige adipose spheroids reveals more profound thermogenic response than 2D cultures

Based on our findings of enriched pathways encompassing oxidative phosphorylation, thermogenesis, and the TCA cycle together with increased expression of the β3-adrenergic receptor in the vascularized adipose spheroids, we next asked if vascularized adipose spheroids represent a more physiological relevant system for induction of thermogenesis. Thus, we utilized the synthetic pan-β-adrenergic agonist Isoproterenol (ISO), which has been shown to upregulate UCP1 and thermogenesis in adipocytes (55), and conducted RNA sequencing of stimulated versus unstimulated vascularized adipose spheroids and 2D cultures.

**Figure 4:**
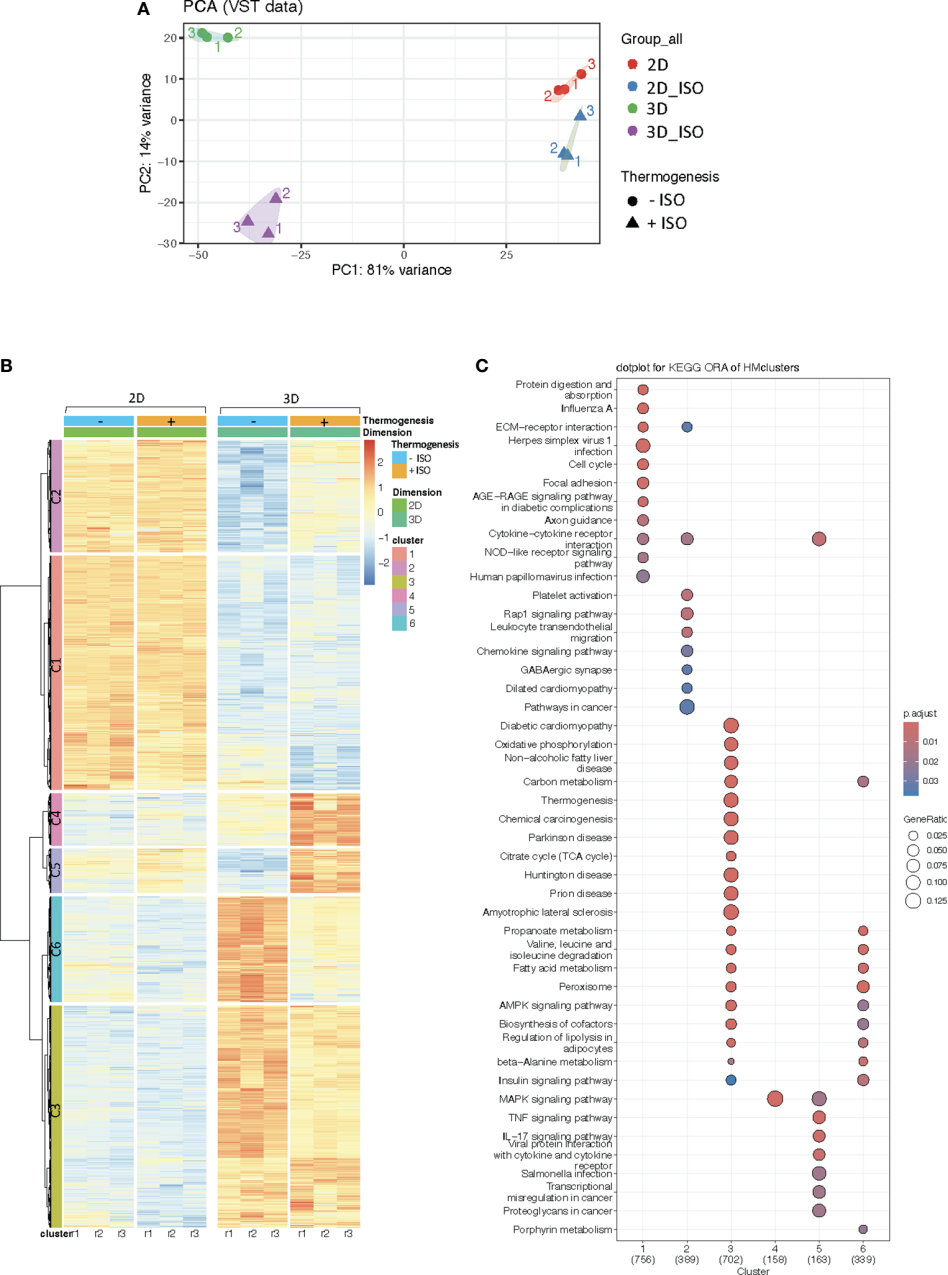
**(A)** Principal Component Analysis (PCA) plot of RNA sequencing data from 2D cultures and vascularized adipose spheroids (3D) +/- Isoproterenol (ISO) stimulation. **(B)** Heatmap of differentially expressed genes (DEG) in 3D vs 2D cultures +/- ISO. **(C)** KEGG Overrepresentation Analysis (ORA) dotplot of pathway enriched genes within each cluster of the heatmap. n = 3. Only genes with a *p*-adjusted value of < 0.05 and a fold change > 1.5 were considered as DEG.

To visualize the complexity of our data, the impact of ISO was assessed using PCA, revealing that stimulation with ISO explains 14% of the variance between the vascularized adipose spheroids, while little transcriptional effects were observed in 2D cultures (**Figure 4A**). Subsequently, heatmap representation provided a visual overview of our DEGs (**Figure 4B**): Cluster (C)1 and C2 contained genes enriched in the 2D cultures, but these genes were mainly ISO-independent, similarly C3 represented genes enriched in the vascularized adipose spheroids, but also ISO-independent (a complete list of differentially expressed genes and gene IDs associated with each cluster are included as **Supplementary Table S2**. On the other hand, C4 and C5 represented ISO-induced genes enriched in the vascularized adipose spheroids, whereas C6 mainly comprised ISO-independent genes enriched in the spheroids. Collectively, the 2D cultures responded to ISO stimulation to a lower degree than the vascularized adipose spheroids, but specific gene sets were subject to adrenergic stimulation in the 3D model system.

ORA analysis demonstrated that genes enriched in C1 and C2 mainly belong to pathways such as protein digestion and absorption, ECM-receptor interaction, focal adhesion, and cytokine-cytokine receptor interaction (**Figure 4C**), resembling the pattern shown in **Figure 2C** for downregulated genes in vascularized adipose spheroids versus 2D cultures. Interestingly, genes in C4 and C5 were involved in pathways such as MAPK (Mitogen-activated protein kinase), cytokine-cytokine receptor interaction, and immune signaling pathways (**Figure 4C**), likely reflecting the notion that MAPK has been implicated in non-shivering thermogenesis and adipocyte beiging. Additionally, C6 comprised genes enriched in pathways mainly related to fatty acid metabolism, insulin signaling, and regulation of lipolysis (**Figure 4C**). This cluster partially overlaps with C3, comprising genes upregulated in the vascularized adipose spheroids and enriched in some of the same pathways as C6, but also in oxidative phosphorylation, thermogenesis, and the TCA cycle (**Figure 4C**), demonstrating similarities to the pathways shown in **Figure 2C** for upregulated genes in the vascularized adipose spheroids.

### 3.5 Comparative transcriptional profiling of thermogenically activated vascularized adipose spheroids and 2D cultures

**Figure 5:**
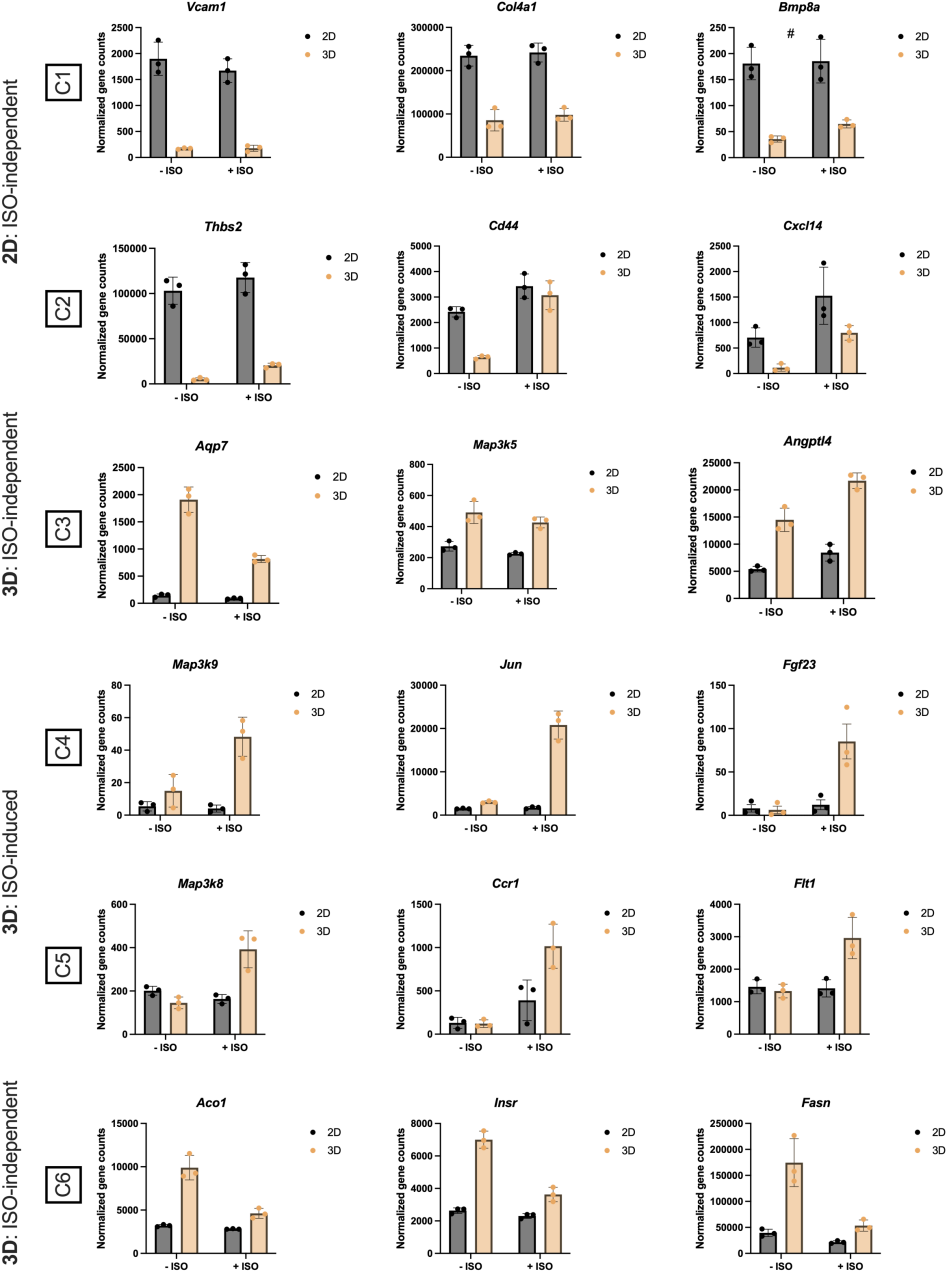
Normalized gene counts derived from the heatmap clusters (C), presented in **Figure 4B**, of 2D and 3D +/- isoproterenol (ISO) stimulated cultures. n = 3. Data is presented as means ± standard deviation (SD), except for *Fgf23*, which is presented as means ± standard error of mean (SEM). Statistics were calculated using unpaired, non-parametric *t*-test followed by Mann-Whitney, # indicates *p* = 0.1.

Examining selected genes from each cluster, we found that *Vcam1* (Vascular cell adhesion molecule 1), *Col4a1* (Collagen type IV alpha 1 chain), *Bmp8a* (Bone morphogenetic protein 8a), and *Thbs2* in C1 and C2 (mainly ISO-independent clusters) (**Figures 5A, B**), associated with pathways like focal adhesion and ECM-receptor interaction, were upregulated in the 2D cultures, corroborating our analysis in **Figures 2C** and **4C** and reflecting the endogenous ECM production and immune processes taking place in the 2D cultures. Whereas *Cd44* and *Cxcl14*, in pathways related to cytokine-cytokine interaction, were upregulated in the 2D cultures, and responded to ISO stimulation. In C4 and C5 (ISO-induced clusters) (**Figures 5D, E**), the expression of *Flt1* (VEGF receptor 1), Ccr1 (CC motif chemokine receptor 1), *Map3k* (Mitogen-activated protein kinase kinase kinase) isoforms and the JNK (c-Jun N-terminal kinase) pathway downstream target *Jun* (Transcription factor AP-1 subunit Jun) were increased in the vascularized adipose spheroids. In addition, the expression of *Fgf23* (Fibroblast growth factor 23) is upregulated solely in the vascularized adipose spheroids in response to ISO. In C6 (ISO-independent cluster) (**Figure 5F**). *Aco1* (Aconitase 1), *Insr* (Insulin receptor), and *Fasn* (Fatty acid synthase) showed unchanged gene expressions in 2D cultures and decreased expressions in vascularized adipose spheroids. *Aco1* regulates iron homeostasis, impacting adipogenic genes such as *Adipoq* (56). INSR mediates insulin signaling in AT, affecting *Fasn* expression.

In C3 (mainly ISO-independent cluster) (**Figure 5C**), expression of *Aqp7* (Aquaporin 7), facilitating glycerol efflux (57), was decreased in vascularized adipose spheroids, while *Angptl4* (Angiopoietin like 4) increased mildly in response to ISO, regulating lipid partitioning (58). Interestingly, *Map3k5* (also known as Ask1), involved in regulation of brown and beige adipocyte function (59), was independently of ISO-stimulation increased in the vascularized spheroids compared to the 2D cultures.

Thus, the vascularized adipose spheroids demonstrated more robust transcriptional effects following adrenergic stimulation than 2D cultures, reflected in the upregulation of known thermogenic pathways such as oxidative phosphorylation, thermogenesis, TCA cycle, but also less explored and gene sets like to MAPK signaling.

### 3.6 Increased oxygen consumption in thermogenically activated vascularized adipose spheroids

**Figure 6:**
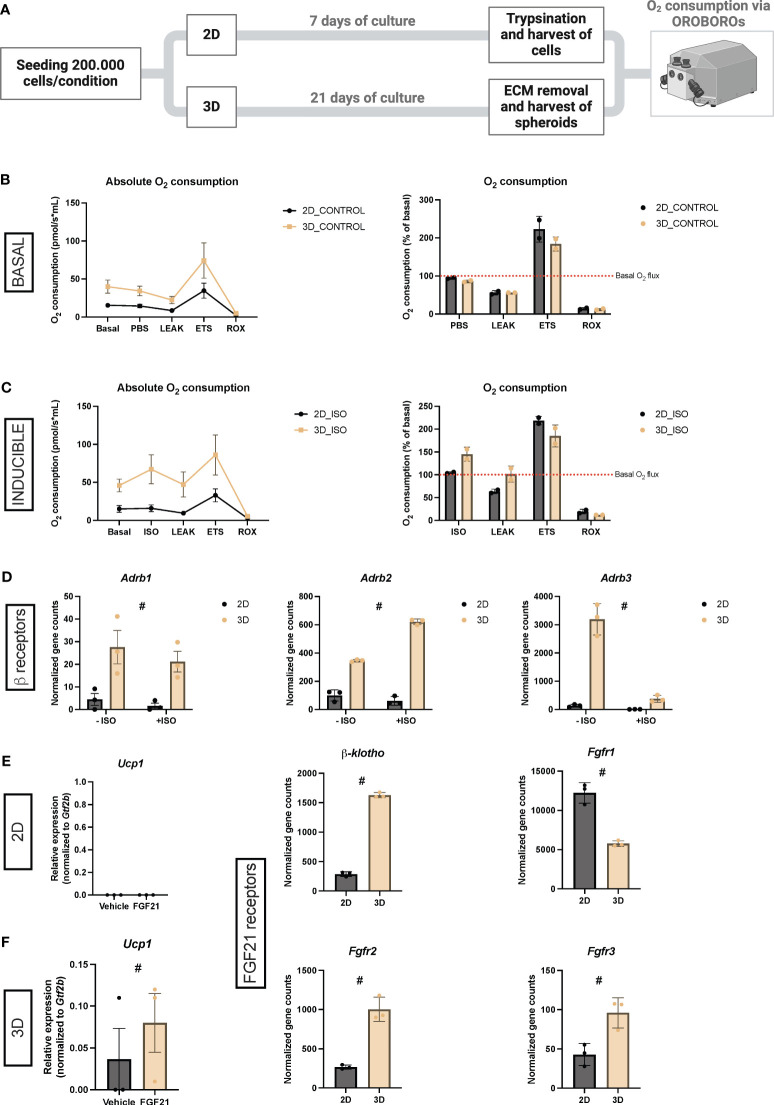
**(A)** Schematic workflow for measuring oxygen consumption via OROBOROs. **(B)** Unstimulated oxygen consumption in 2D cultures and vascularized adipose spheroids (3D) presented as absolute values or % of basal consumption. n = 2. **(C)** Isoproterenol (ISO)-stimulated oxygen consumption in 2D and 3D cultures presented as absolute values or % of basal consumption. n = 2. **(D)** Normalized gene counts of adrenergic β-receptors (*Adrb*) derived from the RNA sequencing data. n = 3. **(E)** Relative mRNA expression of the thermogenic marker *Ucp1* in 2D cultures and vascularized adipose spheroids. The mRNA expressions were normalized to *Gtf2b* expression. n = 3. **(F)** Normalized gene counts of FGF receptors (*Fgfr*) and its co-receptor, derived from the RNA sequencing data. n = 3. Data is presented as means ± standard deviation (SD), except for *Adrb1* and *Ucp1* (3D), which is presented as means ± standard error of mean (SEM). Statistics were calculated using unpaired, non-parametric *t*-test followed by Mann-Whitney, # indicates *p* = 0.1.

Having established that our vascularized adipose spheroids transcriptionally showed enhanced induction of thermogenesis-related genes compared to 2D cultures, we next aimed to assess this functionally by measuring mitochondrial oxygen consumption using Oroboros Oxygraph-2K (O2k Oroboros Instruments). **Figure 6A** represents the workflow of the experiment. The 2D cultures were induced and differentiated until day seven, while the vascularized adipose spheroids were cultured as previously described in **Figure 1A**.

Overall, an increased oxygen consumption was observed in vascularized adipose spheroids compared to 2D cultures after normalization to basal oxygen consumption level (**Figure 6B**), reflecting that the 3D setup has a higher respiratory activity. Addition of oligomycin to inhibit ATP-synthase-linked respiration reduced oxygen consumption to a similar level in both 2D cultures and vascularized adipose spheroids compared to basal consumption, reflecting that coupled respiration in both systems. The addition of FCCP (carbonyl cyanide p-trifluoro methoxyphenylhydrazone), an uncoupler of the oxidative phosphorylation from ATP synthesis, showed that 2D cultures exhibited a 123% increase in maximal oxygen consumption over basal oxygen consumption, 39% more than vascularized adipose spheroids. Furthermore, to evaluate differences in the non-mitochondrial respiration, antimycin A, targeting the electron transport chain, was added, resulting in minimal oxygen consumption for both conditions. This suggests that the 2D cultures had a higher spare respiratory capacity than the vascularized adipose spheroids.

To induce thermogenesis in 2D cultures and vascularized adipose spheroids, the pan β-adrenergic agonist ISO was added to the chambers, resulting in a 45% increase in oxygen consumption over basal consumption for the vascularized adipose spheroids (**Figure 6C**). However, the addition of ISO had almost no effect (4.5% increase) on oxygen consumption in the 2D cultures. Oligomycin decreased oxygen consumption to basal levels in vascularized adipose spheroids and to even lower in 2D cultures. Again, when stimulated with FCCP, 2D cultures exhibited a higher maximal oxidative capacity than vascularized adipose spheroids. The stronger induction of oxygen consumption by ISO-stimulation in vascularized adipose spheroids could be partly explained by the increased expression of the adrenergic β-receptors – *Adrb1, 2* and *3 -* in the vascularized adipose spheroids, when looking at normalized gene counts from our RNA sequencing (**Figure 6D**).

To assess the translatability of our system to study thermogenic effects of naturally occurring hormone, which typically do not cause substantial thermogenic activation of SVF-derived beige fat cells culticated in 2D, we conducted stimulations of 2D cultures and vascularized adipose spheroids with FGF21, a hormone recognized for its capacity to activate thermogenesis and UCP1 in AT (60). As shown in **Figure 6E**, FGF21 stimulation did not influence *Ucp1* mRNA expression in 2D cultures. Conversely, an observable Ucp induction was observed in spheroids, although not statistically significant (**Figure 6E**). This effect might be partly attributed to the increased expression of *Fgf* receptors (*Fgfr*) in the vascularized adipose spheroids showing *Fgfr2*, *Fgfr3*, and the essential FGF co-receptor *β-klotho* all upregulated in the spheroids compared to 2D cultures (**Figure 6F**).

Thus, vascularized adipose spheroids showed increased oxygen consumption and enhanced thermogenic responses, functionally and transcriptionally, compared to 2D cultures.

## 4 Discussion

### 4.1 Adipocyte maturity is influenced by the stromal vascular microenvironment

Adipocyte maturation is influenced by the proximity of adipocytes to the vasculature (16, 36) and the crucial role of the ECM in facilitating adipocyte differentiation and growth signaling (14), which led us to hypothesize that both factors are essential for engineering AT *in vitro*. Despite the fact that adipose-derived endothelial cells synthesize and secrete ECM components themselves (38) and pre-adipocytes grown in suspension cultures or non-adherent surfaces self-assemble into spheroids (61–63), it has been shown in human subcutaneous WAT spheroids (35) that Matrigel-embedding induced vascular sprouts prior to adipocyte differentiation, providing an optimized growth and differentiation niche for the preadipocytes. Indeed, Matrigel-embedded vascularized adipose spheroids (**Figure 1B**) exhibited vascular sprouting, increased cell size (**Figures 1B, C**), and adipogenic gene expression (**Figure 1D**) compared to spheroids not embedded, which is consistent with results reported in the human model (35). This was despite, CD31^+^ endothelial cells were observed in spheroids cultured with and without Matrigel (**Figure 1B**) assisted by mRNA expression for endothelial markers in both (**Figure 1D**). This may be explained by the ability of adipose SVF cells to stimulate endothelial cell morphogenesis into branching vascular networks due to endogenous ECM secretion (64). In consistency with our workflow (**Figure 1A**), it has also been shown that using EGM prior to induction of adipogenesis preserves the SVF-resident endothelial cells and results in vascularized spheroids (34).

### 4.2 Endogenous ECM production as inducer of endothelial, but not metabolic genes

While it is possible to generate 3D adipose cultures without a scaffold and still maintain an enhanced expression of adipogenic genes (61–63), these models are suboptimal for studying angiogenesis. Vascularized adipose spheroids, generated from scaffold-embedded human AT, show increased adipokine secretion, lipid accumulation, glucose and lipolytic responses (35, 65–67). As our results suggested that incorporation of a scaffold supported angiogenic development and thereby enhanced adipogenesis, we hypothesized that our vascularized adipose spheroids were providing an optimized growth niche for adipocytes compared to 2D cultures. Despite the added complexity of including a matrix scaffold in the 3D spheroids, we believe it allows our model to reflect *in vivo* conditions more faithfully by ensuring optimal AT functionality through increased vascularization (68). DEGs in the vascularized adipose spheroids were associated with metabolic pathways, while downregulated genes were linked to ECM formation, cell signaling, and immune responses (**Figure 2B**) although these differential expression changes lack validation at the proteome level given the overall limited cell numbers and protein abundance in spheroids. KEGG and MSigDb ORA analysis highlighted enriched pathways in upregulated genes of the spheroids related to adipogenesis, oxidative phosphorylation, fatty acid metabolism, glycolysis, thermogenesis, and the TCA cycle (**Figures 2C, D**). Notably, expression of endothelial (**Figure 3B**) and ECM (**Figure 3E**) genes were increased in 2D cultures compared to the vascularized adipose spheroids, suggesting a potential positive role of endogenous ECM production, by the SVF, in stimulating endothelial cells (64). Based on our data, we speculate that the endogenous ECM secretion potentially is a stronger inducer of angiogenesis than exogenously added ECM, when looking solely at differences in endothelial and ECM gene expression between 2D cultures and vascularized adipose spheroids. For embryonic stem cells, it has been found that the endogenous ECM secretion is required for cell survival and differentiation, and inhibition of endogenous collagen production leads to a gradual loss of embryonic stem cells, which could not be rescued by addition of Matrigel (69). Moreover, ECM remodeling is a shared process by angiogenesis and inflammation, which directly and indirectly influence both processes. Proteolytic ECM molecules and fragments may act directly as an inflammatory stimulus by influencing immune cell activation and survival, besides proper tissue repair. Additionally, the release of matrix metalloproteinases from inflammatory cells contributes to ECM degradation and release of angiogenic factors such as VEGF and FGF2 (70). This may explain why we in addition also see upregulation of immune related genes in the 2D cultures compared to the vascularized adipose spheroids. While RNA sequencing cannot distinguish cell-specific gene expression, we acknowledge endothelial cell functional heterogeneity and potential overlap with AT transcriptomics from other tissues (71). Future experiments should include single-cell sequencing to identify cell specific subtypes in both 2D cultures and vascularized adipose spheroids.

### 4.3 Insights into adipose physiology: enhanced thermogenesis in vascularized adipose spheroids

To showcase the versatility of our model, we induced thermogenesis with the non-specific β-adrenergic agonist ISO. The vascularized adipose spheroids displayed distinct transcriptional profiles associated with thermogenesis and adipose tissue-related pathways compared to 2D cultures, where mainly focal adhesion and ECM-receptor interaction were dominating (**Figures 4**, **5**). Interestingly, we observed increased expression of *Map3k* isoforms *5*, *9* and *8* in the vascularized adipose spheroids (**Figures 5C–E**). The MAPK cascades are major intracellular signaling pathways, regulating multiple cellular responses, including gene induction of i.e. *Ucp1* via p38, cell death and lipid homoeostasis (72). Although the involvement of MAP3K in controlling brown and beige adipocyte gene expression is less well understood (i.e., the role of isoform 8 and 9), MAP3K5 was recently identified as a regulator of brown and beige adipocyte function by being activated as part of the Protein kinase A-MAP3K5-p38 axis in response to cyclic AMP signaling, thus contributing to the induction of *Ucp1* gene expression (59). We speculate that the expression of *Map3k5* in our vascularized adipose spheroids, although not significant, might similarly contribute to the enhanced induction of thermogenesis compared to 2D cell cultures.

In addition, ISO markedly increased oxygen consumption in spheroids, but minimally affected 2D cultures (**Figures 6B, C**). The enhanced inducibility of thermogenesis in vascularized adipose spheroids may be attributed to increased expression of adrenergic β-receptor subclasses, particularly subclass 3 (**Figure 6D**), which is preferentially expressed in adipocytes (55) and involved in thermogenesis of brown and beige adipocytes (1). While ISO is a synthetic, non-selective inducer of thermogenesis and might activate other cell types in the SV microenvironment, future experiments should explore more specific inducers like the highly selective β3-adrenoreceptor agonist CL316,243 (73). Nonetheless, FGF21, a natural occurring adipokine, proven to increase BAT thermogenesis and induce browning of WAT (60, 74) increased UCP1 mRNA expression in vascularized adipose spheroids, but had no detectable effect in 2D cultures. These results highlight the versatility of the vascularized adipose spheroids in resembling physiological responses related to thermogenesis observed *in vivo*, emphasizing the potential of the spheroids for studying beige adipocyte activation and related metabolic pathways.

### 4.4 Advancing adipose tissue research: a novel, universal workflow for vascularized adipose spheroids

*In vitro* monolayer cultures lack the complexity observed in real-life tissues, and 2D cultures, while common, fail to replicate *in vivo* microenvironments and ECM interactions (75). Consequently, 3D cultures are crucial for engineering *in vivo* tissue microenvironments. The choice of 3D model depends on the research question, and the high versatility of these models often leads to unique setups for each question. This diversity makes it challenging to compare various 3D models, as they are rather customized than universal. Our easy-to-follow, universal workflow for the assembly of vascularized adipose spheroids from the iWAT depot serves as a novel, versatile 3D platform for studying adipose metabolism *in vitro*. Indeed, future experiments should include more replicates to obtain statistical significance of the gene expressions provided in this study. This especially applies to the OROBOROS experiment. Moreover, differences in oxygen consumption levels must be carefully interpreted as the SVF seeded in 2D cultures and the vascularized adipose spheroids was counted at the beginning of the experiment, but not the end, which may lead to differences in cell numbers between the two conditions which is unlikely, though, given that adipogenic differentiation leads to cell cycle arrest in 2D and spheroids.

## 5 Conclusion

We demonstrated that the vascularized adipose spheroids functionally and transcriptionally show superiority over 2D counterparts, as we obtained induction of vascular sprouts, enhanced expression of genes associated with adipogenesis and metabolism, and increased oxygen consumption. This suggests that the adipocytes within the spheroids are more mature, likely influenced by the 3D microenvironment that allow the adipocytes to be in contact with other cells from the SV microenvironment.

As tissue engineering evolves, we speculate that transplantation of our beige thermogenic vascularized spheroids could enhance *in vivo* thermogenesis, given their potential to develop vascular interconnections with surrounding tissue blood vessels (34). Although similar vascularized organotypic adipocyte setups from human sources (35) clearly showed a tight transcriptional and functional connection between vascular outgrowth (sprouting) and degree of adipogenesis, the degree of differentiation at the single adipocyte level could not be assessed in this study. Future work will therefore focus on the nature of (vasculature)-derived pro-adipogenic factors, metabolites and micro-vesicles and to, at the single adipocyte level, quantify the magnitude of adipogenic differentiation, lipid accumulation and adipokine secretion.

## Data availability statement

The data presented in the study are deposited in the Gene Expression Omnibus (GEO) repository, accession number GSE261267.

## Ethics statement

Mouse experiments were conducted with permission from the Danish Animal Experiments Inspectorate, reference number: 2023-15-0201-01544. The study was conducted in accordance with the local legislation and institutional requirements.

## Author contributions

JK: Conceptualization, Funding acquisition, Project administration, Supervision, Writing – review & editing. LD: Conceptualization, Formal analysis, Methodology, Project administration, Resources, Visualization, Writing – original draft, Writing – review & editing. CH: Conceptualization, Methodology, Writing – review & editing. VG: Data curation, Formal analysis, Visualization, Writing – review & editing. SL: Data curation, Formal analysis, Investigation, Writing – review & editing. SL: Conceptualization, Funding acquisition, Project administration, Resources, Writing – review & editing. MFE: Data curation, Formal analysis, Investigation, Writing – review & editing. NS: Methodology, Writing – review & editing. HT: Supervision, Writing – review & editing.

## Supplementary material

Supplementary Table 1 Contains sequences for quantitative RT-PCR (qPCR) primers used in this study.

Supplementary Table 2 Contains normalized count tables, and differential expression of groups shown in **Figure 4**.
