# Supplementary material for: Mouse vascularized adipose spheroids: an organotypic model for thermogenic adipocytes
Source: Front Endocrinol (Lausanne). 2024 Jun 25;15:1396965. doi: 10.3389/fendo.2024.1396965 (PMC11231189; doi:10.3389/fendo.2024.1396965)
Supplement: Supplementary Table 1 — Contains sequences for quantitative RT-PCR (qPCR) primers used in this study. [file Table_1.docx]

| *Gene* | *Forward* | *Reverse* |
| --- | --- | --- |
| *Gtf2b* | GTGGGATCTGAATGGAGAACTT | CCTGTACCCTTGCCAATCAT |
| *Adipoq* | ACTTGTGCAGGTTGGATGGC | CCCTTCAGCTCCTGTCATTCC |
| *Fabp4* | AGGCCTGGCCTTTGACTTAGA | TGAGGCAGTTTGACCATTTTATTCT |
| *Cdh5* | TGAATCGCTGCCCCACTATG | GTGTTAGCATCGACCCCGAA |
| *Rbp7* | GGAATCTTCTCAGCAGCGAC | GATGGTGAAGGAGTCCCCATT |
| *Ucp1* | ACACTTTGGAAAGGGACGAC | GCAAAACCCGGCAACAAGAG |

***S.Table 1.*** *Primer sequences for qPCR.*
